# MSCFS: inferring circRNA functional similarity based on multiple data sources

**DOI:** 10.1186/s12859-021-04287-1

**Published:** 2021-07-16

**Authors:** Liang Shu, Cheng Zhou, Xinxu Yuan, Jingpu Zhang, Lei Deng

**Affiliations:** 1 School of Computer Science and Engineering, Central South University, Lushangnan Road, Changsha, China; 2 Department of Chemical and Life Science Engineering, Virginia Commonwealth University, Richmond, VA 23284 USA; 3 School of Computer and Data Science, Henan University of Urban Construction, Longxiang Road, Pingdingshan, 467000 China

**Keywords:** CircRNA functional similarity, Multiple data sources, Multiple representations

## Abstract

**Background:**

More and more evidence shows that circRNA plays an important role in various biological processes and human health. Therefore, inferring the circRNA’s potential functions and obtaining circRNA functional similarity has become more and more significant. However, there is no effective approach to explore the functional similarity of circRNAs.

**Methods:**

In this paper, we propose a new approach, called MSCFS, to calculate the functional similarity of circRNA by integrating multiple data sources. We combine circRNA-disease association, circRNA-gene-Gene Ontology association, and circRNA sequence information to explore the functional similarity of circRNA. Firstly, we employ different learning representation methods from three data sources to establish three circRNA functional similarity networks. Then we integrate the three networks to obtain the final circRNA functional similarity.

**Results:**

We utilize circRNA–miRNA association similarity and circRNA co-expression similarity to evaluate the performance of MSCFS. The results show a positive correlation with miRNA association ($R=0.213$) and circRNA co-expression similarity ($R=0.8991$). Finally, we construct a circRNA functional similarity network and perform case analysis. The result shows our method can be applied to infer new potential functions of circRNA and other associations.

**Conclusions:**

MSCFS combines multiple data sources related to circRNA functions. Correlation analysis and case analyses prove that MSCFS is a useful method to explore circRNA functional similarity.

## Background

Circular RNAs, a class of endogenous non-coding RNAs, are characterized by their covalently closed-loop structures without a 5$^{\prime }$ cap or a 3$^{\prime }$ Poly A tail [1]. Sanger et al. [2] first found CircRNAs in 1976. However, the circRNAs were thought to be splicing artifact; and were continuously considered as “junk” RNAs for about two decades [3]. More and more researches have corroborated that circRNAs play an essential role in many cell activities, affecting arteriosclerosis and participating in mRNA expression variable splicing regulation [4–9]. In recent years, circRNAs have been identified as biomarkers and therapeutic targets for various acute diseases. CircRNA is associated with a variety of chronic diseases, such as lung cancer, Alzheimer’s disease, diabetes, cardiovascular disease and other [10, 11]. The emerging experimental results have corroborated that circRNA molecules have abundant miRNA binding sites and act as miRNA sponges in cells to releasing the inhibitory effect of miRNA on their target genes and improving the expression level of target genes [12–14]. Identifying the targets of circRNAs helps to understand the functions of circRNAs. Several efforts have been developed to identify circRNA targets [15–17]. For example, Lin et al. [17] designed Analysis of common targets (ACT) to facilitate the identification of potential circRNA targets.

Functional similarity can be defined as an association, such as co-expression similarity, co-Gene Ontology (GO) term similarity, co-similar disease similarity, and co-literature similarity [18]. Analogous to the methods of studying the functional similarity of microRNA, Wang et al. [19] obtained the functional similarity of miRNA through the DAG of disease and microRNA-disease association. The Gene Ontology (GO) project provides the most comprehensive resource currently available for computable knowledge regarding the functions of genes and gene products. Gene Ontology provides the logical structure of the biological functions (‘terms’) and their relationships to one another, manifested as a directed acyclic graph [20]. Yang et al. [21] obtained the functional similarity of microRNA by calculating GO semantic similarity and miRNA-GO association. There are many methods to acquire the semantic similarity of GO, such as the measures proposed by Resnik et al. [22], Jiang et al. [23], Lin et al. [24], Wang et al. [25], and Wu et al. [26]. Obtaining the functional similarity of RNA can also be obtained through sequence information. Sequence similarity can be calculated by methods such as K-mer [27] or LSTM [28].

**Fig. 1:**
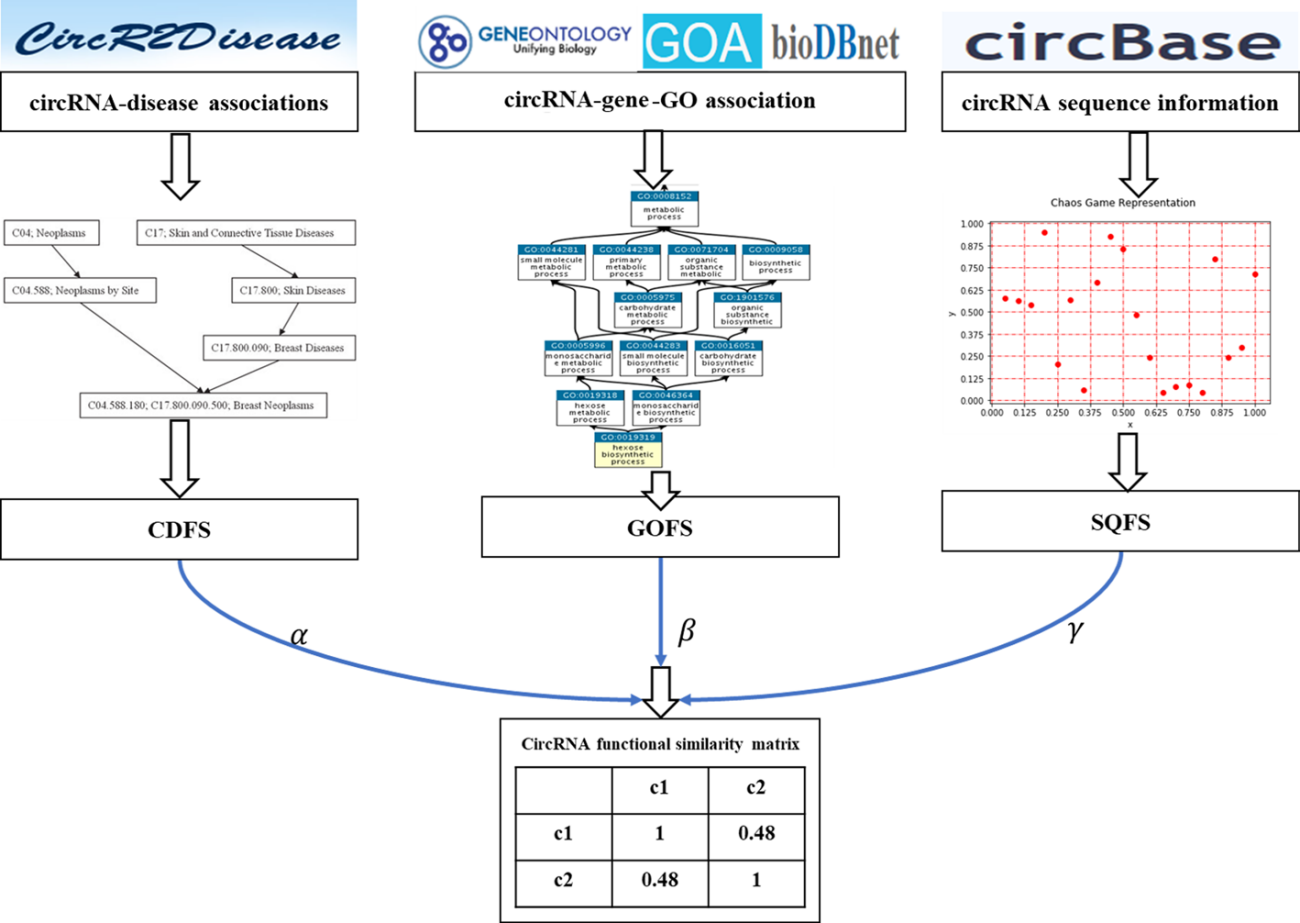
Overview of MSCFS demonstrating the basic ideas of measuring circRNAs functional similarity

However, there is no valid method to calculate the functional similarity of circRNA, and a single circRNA data source can’t effectively explore the circRNA functional similarity. In this paper, we propose a novel method called MSCFS by integrating multiple biological data sources to calculate the functional similarity between circRNAs. Firstly, we obtain the circRNA functional similarity matrix by using the DAG graph and association information of the disease. Secondly, we construct the corpus through circRNA-gene-GO associations and GO annotations and employ word2vec to obtain the circRNA functional similarity matrix. Thirdly, we adopt chaos game representation to get circRNA functional similarity by circRNA sequence information. Finally, the circRNA functional similarity is obtained by integrating the three networks. The results show that MSCFS is efficacious and accurate, and it can infer the potential functions of circRNA. The flowchart of our proposed model is shown in Fig. 1.

## Methods

### Dataset

We downloaded the MeSH descriptor from the National Library of Medicine (http://www.nlm.nih.gov/) [29]. MeSH descriptors are divided into 16 categories: category A is anatomical terms, category B is organisms, category C is diseases, category D is drugs and chemicals, etc. Then, we obtained the relationship of various diseases based on DAG diseases from the MeSH descriptor of category C.

Many benchmark databases contain circRNA-disease association data, such as circR2Disease [30], circRNADisease [31], circFunBase [32], and Circ2Disease [33], which contain experimentally verified associations between circRNAs and diseases. We utilize circR2Disease as the benchmark data set. Circ2Disease is a database that can manually manage human circRNA supported by experiments and provide the association between circRNA and human diseases. We obtained 418 confirmed circRNA-disease associations consisting of 365 circRNA and 71 diseases after removing the circRNAs in which the gene symbol could not be found.

We downloaded the Gene Ontology (GO) in OWL format from the Gene Ontology Consortium (GOC) [34] and GO annotations in the Gene Ontology Annotation (GOA) Database [35]. We used the OWL API version 4.2.6 to process the GO in OWL format.

We extracted 321 genes associated with circRNA and the circRNA sequence information from the circBase [36]. We obtained 7321 GO-gene associations from multiple versions of the database bioDBnet [37].

### Overview of MSCFS

In this article, we combine the three data sources of circRNA to calculate the functional similarity of circRNA. Specifically, we obtain three circRNA functional similarity matrices from the circRNA-disease association, circRNA-gene-GO association, and circRNA sequence information. Finally, we integrate three networks to obtain the final circRNAs functional similarity, and the formula is as follows:

1 $$\begin{aligned} \left\{ \begin{array}{lr} \mathrm {FS}=\alpha * \mathrm {CDFS}+\beta * \mathrm {GOFS}+\gamma * \mathrm {SQFS} \\ \alpha +\beta +\gamma = 1 \end{array} \right. \end{aligned}$$

where CDFS, GOFS, SQFS are circRNA functional similarity matrices obtained through circRNA-disease association, circRNA-gene-GO association, and circRNA sequence information, respectively. $\alpha$, $\beta$, and $\gamma$ are the weighting coefficients of the three networks severally.

### Functional similarity based on circRNA-disease association

**Fig. 2:**
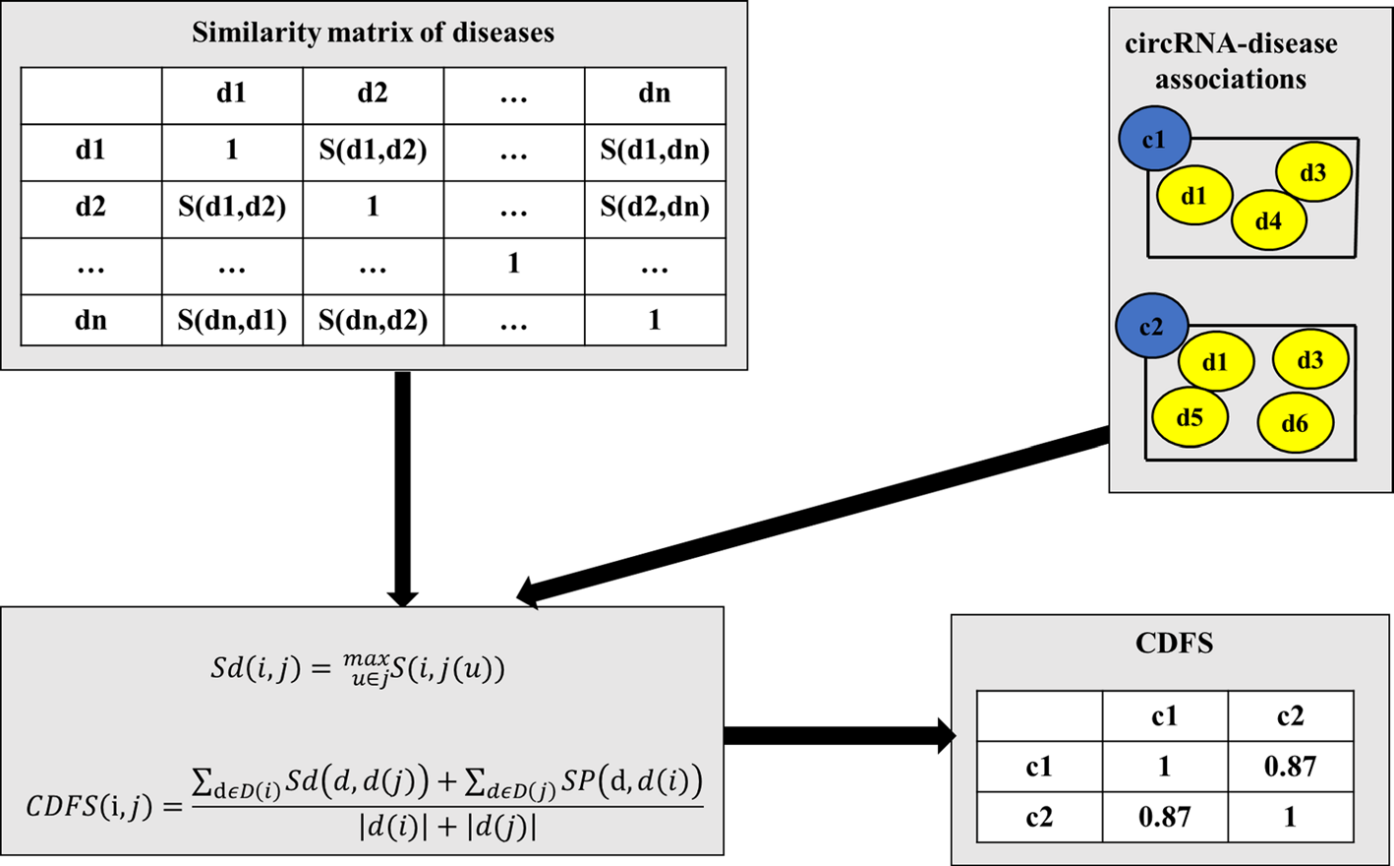
Calculating circRNA functional similarity based on circRNA-disease association

Genes with similar functions are known to be associated with similar diseases. A structure of a directed acyclic graph (DAG) can represent the relationship between different diseases. Therefore, we can calculate the functional similarity of circRNA through circRNA-disease association. The process is shown in Fig. 2.

In the MeSH database, the relationship between diseases is described in the form of a directed acyclic graph (*DAG*), where nodes represent diseases and edges represent relationships between diseases. Given a disease *D*, we have defined a DAG graph $DAG_{D}=\left( D, T_{a}, E_{a}\right)$ based on the other diseases it is associated with and related edges, where $T_{a}$ is the set of ancestor nodes containing itself, and $E_{a}$ is the set of corresponding edges connecting these diseases. If disease *d* is in the *DAG*, its contribution to disease *A* can be calculated as follows:

2 $$\begin{aligned} \left\{ \begin{array}{ll} D_{D}(D)=1 \\ D_{D}(d)=\max \left\{ \Delta * D_{A}\left( d^{\prime }\right) \mid d^{\prime } \in { children\ of } \ d \, \right\} \ if \ d \ne A \end{array} \right. \end{aligned}$$

where $\Delta$ is the semantic contribution factor of disease d and its child nodes. In DAG, the semantic value of disease D itself is defined as 1. Therefore, through the following formula, we calculate the semantic value *DV*(*D*) of disease *D*:

3 $$\begin{aligned} {\mathrm {DV}}({\mathrm {D}})=\sum _{d \in T_{A}} D_{A}(d). \end{aligned}$$

Here, we assume that the more *DAG* shared parts of the two diseases, the higher the semantic similarity, so according to the position of the two diseases in the *DAG* graph and the semantic relationship with the ancestral diseases, the formula for calculating the semantic similarity of the two diseases *M* and *N* is as follows:

4 $$\begin{aligned} S(M, N)=\frac{\sum _{d \in T_{M \cap } T_{N}}\left( D_{M}(d)+D_{N}(d)\right) }{D V(M)+D V(N)}, \end{aligned}$$

where $D_{M}(d)$ is the semantic value of disease d related to disease *M*, and $D_{N}(d)$ is the semantic value of disease d related to disease *N*.

5 $$\begin{aligned} S d(d, D T)=\max _{u \in D T} S(d, D T(u)), \end{aligned}$$

where *DT* is a group of diseases, *u* is any disease in *DT*. After obtaining the semantic similarity of disease combinations, we use circRNA-disease correlation to obtain the functional similarity of circRNA, CDFS.

6 $$\begin{aligned} {CDFS}(i, j)=\frac{\sum _{d \in D(i)} S d(d, d(j))+\sum _{d \in D(j)} S d(d, d(i))}{|D(i)|+|D(j)|}, \end{aligned}$$

where *CDFS*(*i*, *j*) is the similarity between the *i*th circRNA and the *j*th circRNA, *D*(*i*) is the *i*th circRNA associated disease set.

### Functional similarity based on circRNA-gene-GO association

Onto2Vec is a measure that combines formal ontology axioms and annotation axioms in ontology metadata to generate a vector representation of biological entities in the ontology [38]. Gene Ontology contains the representation of the essence of the knowledge system in the field of biology. Ontologies are usually composed of a set of categories (or terms or concepts) with relationships between them. In order to explore the functional similarity of circRNA, we use the circRNA-gene-GO association. We add circRNA as new entities and apply the $has{-function}$ relationship to connect them with their functions to generate a corpus. Then we use Onto2Vec to generate a vector representation for each class (using a corpus to only be based on axioms), and further, generate a joint representation of circRNA and classes (using a corpus-based axiom and circRNA and its annotations).

**Fig. 3:**
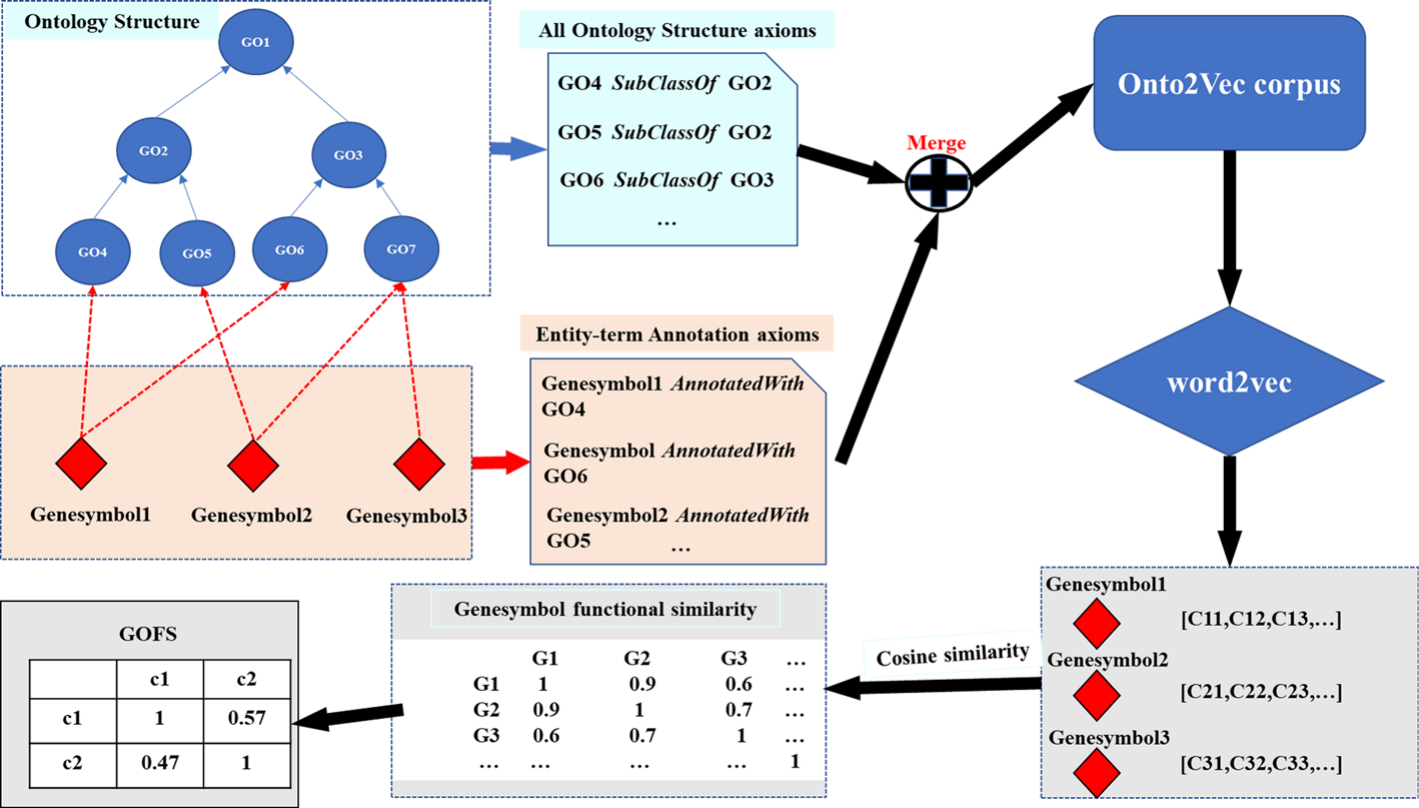
Using word2vec to get circRNA functional similarity based on circRNA-gene-GO association and GO annotations

In the end, we constructed 230,699 corpus, with 50,409 categories, using the Skip-gram model in word2vec. Word2Vec is a set of neural network-based tools that can generate vector representations of words from a large corpus. There are two models: the continuous bag of word (CBOW), which uses a context to predict a target word, and the Skip-gram model that tries to maximize the classification of a word based on another word from the same sentence. Figure 3 shows the flow of this section.

The Skip-gram model is chosen because the Skip-gram model generates higher quality rare word representations in the corpus. The Skip-gram model learns more detailed word vectors and has a large number of low-frequency words in the corpus to produce high-quality representations of all biological entities occurring in our large corpus, including uncommon ones. Given a set of training word sequences ${w}_1, {w}_2\ldots , {w}_N$, Skip-gram the goal is to maximize the following average logarithmic likelihood values:

7 $$\begin{aligned} \frac{1}{N} \sum _{t=1}^{N} \sum _{-s \le i \le s, j \ne 0} \log p\left( \omega _{t+j} \mid \omega _{t}\right) , \end{aligned}$$

where *s* means the size of the training context, *N* means the size of the set of the training words, and ${w}_i$ is the *i*th training word in the sequence. In our research, the parameters of word2vec used are shown in Table 1.

**Table 1:** Parameters of Word2Vec model

Parameter	Definition	Default value
*sg*	Choice of training algorithm (sg = 1:skip gram; sg = 0:CBOW)	1
$min\_count$	Words with frequency lower than this value will be ignored	1
*size*	Dimension of the obtained vectors	200
*window*	Maximum distance between the current and the predicted word	10
*iter*	Number of iterations	5
*negative*	Whether negative sampling will be used and how many ’noise words’ would be drawn	4

Through the training, we get the similarity of the genes, and then through the circRNA-gene relationship GOFS, the calculation formula is as follows:

8 $$\begin{aligned} {\mathrm {GOFS}}(i, j)=\frac{\sum _{g \in G(i)} S(g, g(j))+\sum _{g \in G(j)} S(g, g(i))}{|g(i)|+|g(j)|}, \end{aligned}$$

where *g*(*i*) is the set of genes associated with the *i*th circRNA, and *g*(*j*) is the set of genes associated with the *j*th circRNA.

### Functional similarity based on circRNA sequence

**Fig. 4:**
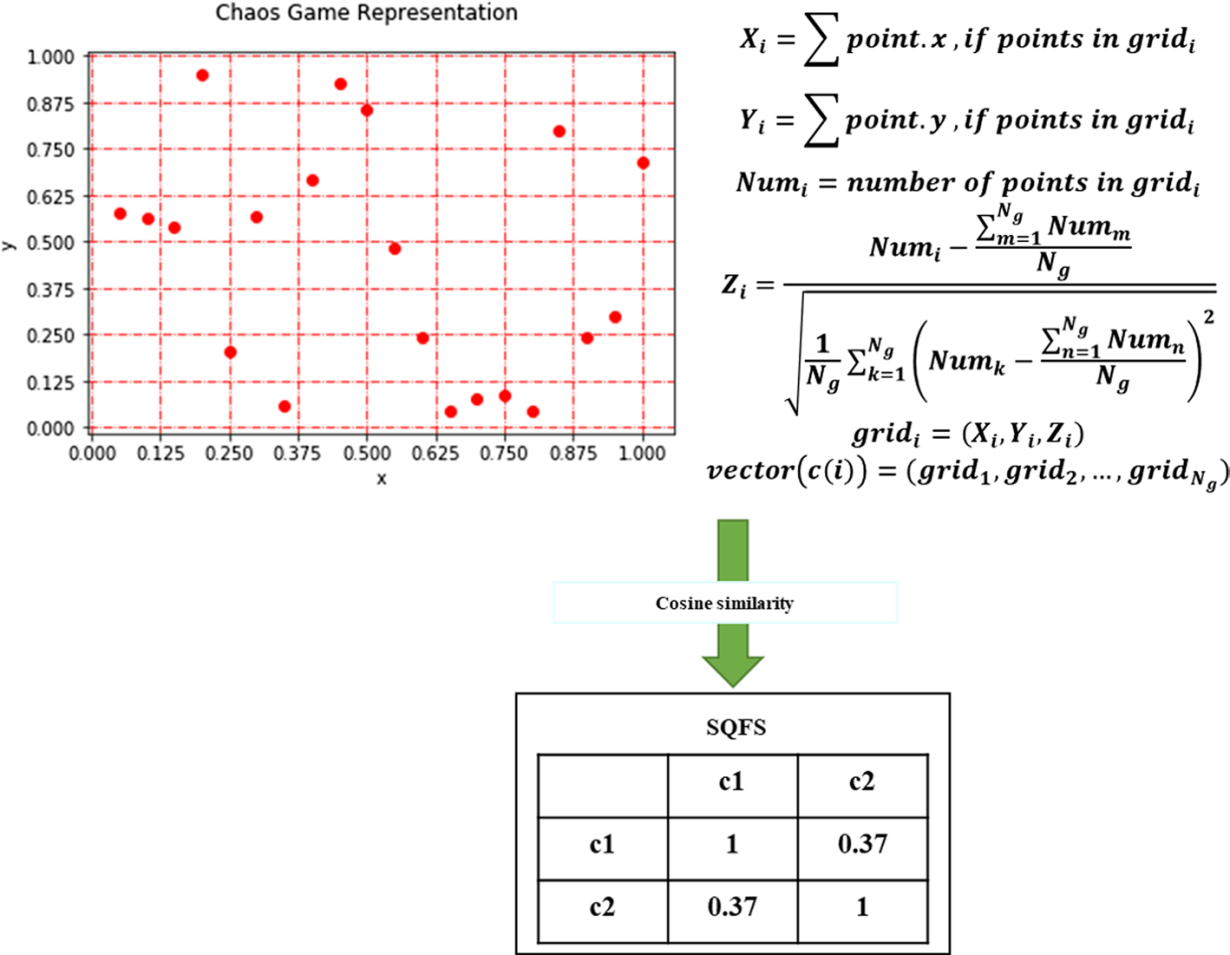
Utilizing Chaos game representation to obtain circRNA functional similarity though circRNA sequence information

Different from the K-mer [27], PSSM method [39], chaos game representation [40] combines position information and nonlinear relationship to obtain vector representation of sequences. Finally, the Pearson correlation is used to quantify their correlation. The advantage of the algorithm is that the original information of the sequence is completely restored in the coordinate system, and the information will not be lost in the mapping. Secondly, the position information will be retained as a mapping. Figure 4 shows the workflow of this section.

The position of each nucleotide in the plane:

9 $$\begin{aligned} P_{i}=0.5*\left( P_{i-1}+S_{i}\right) \quad i=1\ldots N , \end{aligned}$$

where ${P}_0$ is any given starting point $(P_{0}=(0.5,0.5))$, *N* represents the length of the sequence. ${S}_i$ represents the *i*th nucleotide in the sequence, which corresponds to the fixed vertex coordinates of $A=(0,0)$, $C=(1,0)$, $G=(1,1)$ and $U=(0,1)$ respectively.

In this way, the CGR graph is transformed into a $N_{g}$ grid $\left( N_{g}=2^{s} \times 2^{s}, \mathrm {s}=3\right)$ digital matrix, which is called the frequency matrix of CGR graph (FCGR). And grid can be represented as follows:

10 $$\begin{aligned} {grid}_{i}=\left( X_{i}, Y_{i}, Z_{i}\right) \end{aligned}$$

We use the x-axis, y-axis direction and their digital features to construct the feature vector of the sequence, the calculation formula is as follows: the abscissa point.x and ordinate point.y in each grid are accumulated respectively to quantify position information.

11 $$\begin{aligned} \left\{ \begin{array}{ll} X_{i}=\sum {point.x }&{} \quad {if \ points \ in\ grid }_{i}\\ Y_{i}=\sum {point. y}&{} \quad {if \ points \ in\ grid }_{i} \end{array}. \right. \end{aligned}$$

Then, we obtain the *z*-scores of each grid $Z_{i}$ to quantify potential features.

12 $$\begin{aligned} \left\{ \begin{array}{ll} Z_{i}=\frac{ {Num}_{i}-\frac{\sum _{m=1}^{N_{g}} N u m_{m}}{N_{g}}}{\sqrt{\frac{1}{N_{g}} \sum _{k=1}^{N_{g}}\left( {Num}_{k} -\frac{\sum _{n=1}^{N_{g}} N u m_{n}}{N_{g}}\right) ^{2}}}\\ \\ { Num }_{i}= number \ of \ points\ in \ grid_{i} \end{array} \right. \end{aligned}$$

Finally, each grid can be represented as three attributes, and we fused the attributes to construct the vectors *vector*(*c*(*i*)) to define the sequence functional similarity of circRNAs *SQFS*(*c*(*i*), *c*(*j*)) by Pearson correlation coefficient. Where *c*(*i*) represents the *A* *i*th cricRNA.

13 $$\begin{aligned} \left\{ \begin{array}{ll} {SQFS(c(i),c(j))}=\frac{ {vector}({c(i))} \cdot {vector}({c(j))}}{\Vert {vector}({c(i))}\Vert \Vert {vector}({c(j))}\Vert }\\ { vevtor }(c(i))=\left( {grid}_{1}, {grid}_{2}, \ldots , {grid}_{\mathrm {N}_{g}}\right) \end{array}, \right. \end{aligned}$$

where *vector*(*c*(*i*)) means the sequence feature vector of the *i*th circRNA, ${vector(c(i))}\cdot {vector(c(i))}$ is the dot product of *vector*(*c*(*i*)) and *vector*(*c*(*i*)).

## Results

### Parameters selection

circRNA can be used as the sponge of microRNA to play a role in biological processes [12]. We downloaded circRNA–miRNA association data from the starbase [41]. There are 267 types of circRNAs that match the 365 types of circRNA we calculated. We obtained 267 pairs of circRNA–miRNA association similarities through the Jaccard similarity method, which were compared with the functional similarities we calculated. Denote *CMS* as the circRNA–miRNA similarity matrix, and its entry *CMS*(*i*, *j*) can be obtained by the following formula:

14 $$\begin{aligned} CMS\left( i,j\right) =\frac{\left| {CM}_i\cap {CM}_j\right| }{\left| {CM}_i\cup {CM}_j\right| }, \end{aligned}$$

where ${CM}_i$ is the set of microRNAs associated with the *i*th circRNA, and ${CM}_j$ is the set of microRNAs associated with the *j*th circRNA.

**Fig. 5:**
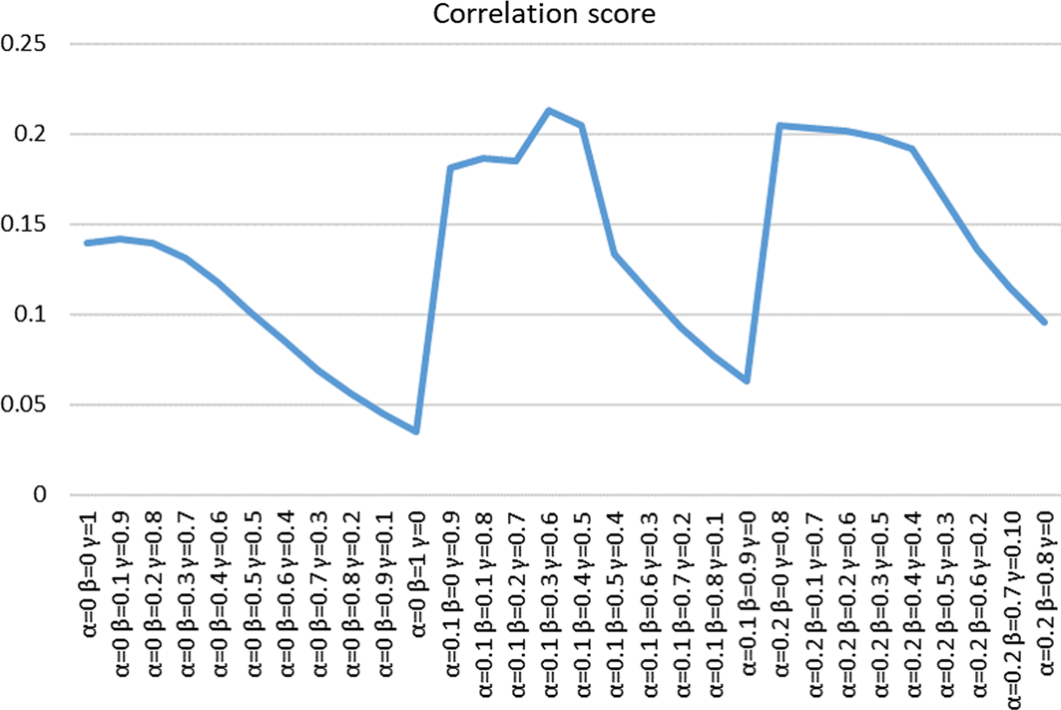
Parameters selection

**Fig. 6:**
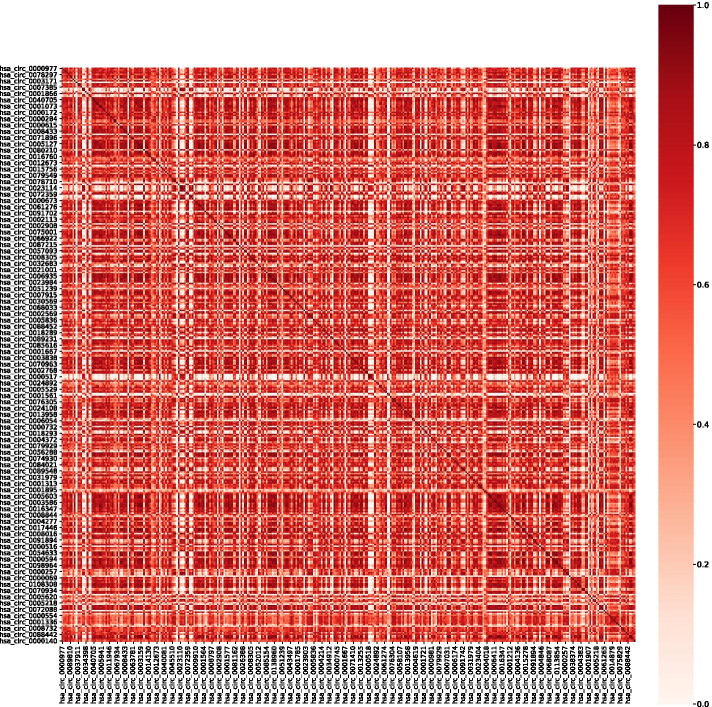
CircRNA functional similarity heatmap shows the functional similarity of 365 kinds of circRNAs

We set the parameter step size to 0.1. Because circRNA has little data associated with diseases, we set $\alpha$ the value range from 0 to 0.2, and the value range for $\beta$ and $\gamma$ from 0 to 1.We used the grid search method to obtain the optimal parameters through 30 sets of experiments and selected two groups for display. The results are shown in Fig. 5. The experimental results with parameters of 0.1, 0.4, 0.5 and 0.1, 0.3, 0.6 are $({R=0.205}, {P=8.2e^{-3})}$, $({R=0.213}, {P=4.6e^{-4}})$. The results prove that our circRNA functional similarity is related to microRNA similarity. We selected the optimal value of the parameter $\alpha ,\beta ,\gamma$ are 0.1, 0.3, 0.6. Figure 6 shows the heat map of circRNA functional similarity calculated with parameters of 0.1, 0.3 and 0.6.

### CircRNA functional similarity is correlated with expression similarity

CircRNAs with semblable functions squint towards participate in semblable biological processes and interact with semblable cellular components. To verify circRNAs with similar functions may squint towards having similar expression profiles, we seeked the relationship between circRNA functional similarity calculated by MSCFS and expression similarity. In this study, we used the absolute Pearson’s correlation coefficient (PCC) to measure circRNA expression similarity.

**Fig. 7:**
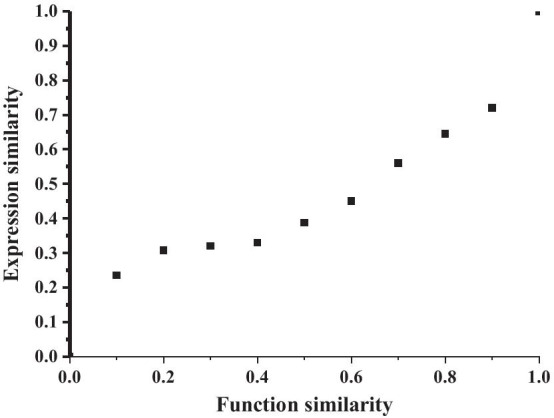
The relationship between circRNA MSCFS functional similarity and their expression similarity

We finally obtain circRNA expression profiling data from Peng et al.’s work [42], which consists of expression profiles of 2932 circRNAs. Then, we calculated the conducted a comprehensive analysis of circRNA expression in papillary thyroid carcinoma PCC score as the co-expression similarity of each pair of circRNA expression profiles and obtained the co-expression similarity of 8049 pairs of circRNAs, and then performed the correlation analysis of circRNA expression similarity and circRNA functional similarity. As a result, the functional similarity of circRNA confirmed positive correlation with circRNA co-expression similarity (${R=0.076, P=9.05e^{-4}}$, Pearson correlation). We grouped 8049 pairs of circRNAs into different groups according to functional similarity in steps of 0.1 and calculated the average expression similarity and functional similarity of each group. Clearly, the functional similarity of circRNA is positively correlated with the expression similarity (${R=0.8991, P=9.73e^{-4}}$, Fig. 7). Results inform that circRNA functional similarity obtained by our method is correlated with circRNA expression similarity, which is well known to be associated with circRNA functional similarity.

### A circRNA functional similarity network

**Fig. 8:**
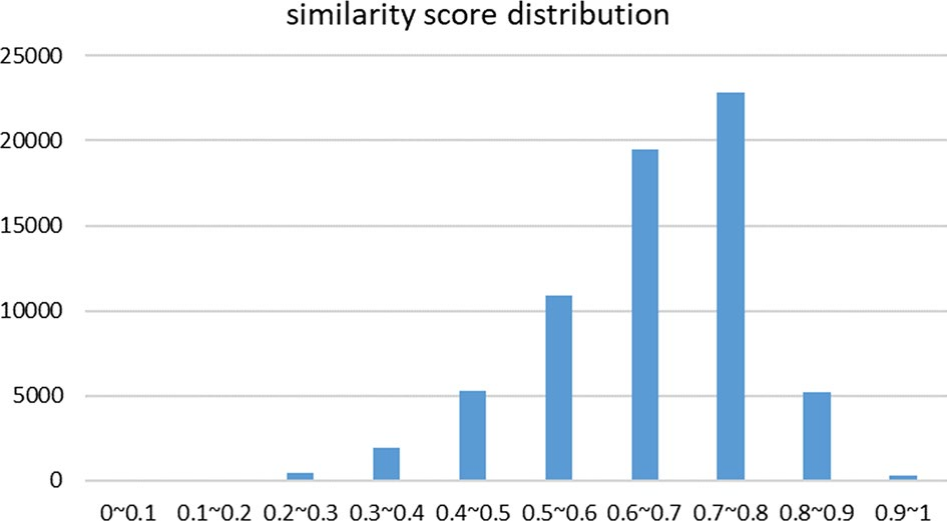
The circRNA funtional similarity score distribution

**Fig. 9:**
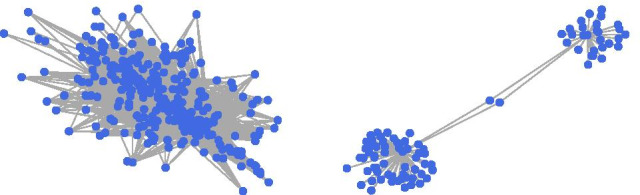
A circRNA functional network based on the functional similarity of MSCFS. Each node and edge connecting any two nodes (circRNAs) indicates that the functional similarity of the two circRNAs is equal to or greater than the similarity cutoff value

Figure 8 shows the distribution of circRNA functional similarity scores. We have structured a partial graph of the circRNA network with a threshold of 0.7 (Fig. 9). Some circRNAs are less associated with other circRNAs, while some circRNAs are more associated with other circRNAs. We can do more research on those circRNAs that are more related and explore the potential functions of circRNAs related to them.

### Case study

To verify our results, we executed case analysis on the circRNA function annotation in the CircFunBase database. CircFunBase is a web-accessible database that aims to provide a high-quality functional circRNA resource, including experimentally validated and computationally predicted functions [32].

Amongst the 365 kinds of circRNAs, hsa_circ_0000140 has the highest correlation score with hsa_circ_0001946 in other 364 circRNAs. In the CircFunBase database, the functional annotations of these two circRNAs are related to gastric cancer. Then we select circRNA pairs with high similarity scores for analysis. For example, the functional similarity score of hsa_circ_0043278 and hsa_circ_0006220 is 0.82, and it can be found in the CircFunBase database that both are related to hypertension [differential expression (hypertensive patients and healthy controls)]. The functional similarity score of hsa_circ_0005927 and hsa_circ_0138960 is 0.83, both of which are related to gastric cancer. Through case analysis, we can know the practicality and accuracy of the MSCFS method.

## Discussion and conclusion

CircRNAs have peculiar biological structures and have proven to play essential roles in biological processes and human health. Inferring the functional similarity of circRNAs can help analyze the function of circRNAs and predict the association of circRNA-disease. However, due to the lack of functional annotations for circRNAs in public databases, it is not straightforward to calculate the functional similarity of circRNAs using existing single data sources.

This paper proposes a new algorithm, MSCFS, to calculate the functional similarity of circRNA by integrating multiple circRNA associated biological data. The results showed that the circRNAs associated with the same miRNA have a high similarity score. The circRNA co-expression similarity was positively correlated with our calculated results. We also found that circRNAs with a high similarity score were also similar in the function of the disease. By calculating the functional similarity of circRNAs, we can explore more potential functions and associations of circRNAs.

We have integrated multiple biological data sources related to circRNAs, and the results will be somewhat biased due to the quality of some data sources. In the future, we will integrate more and more reliable data to further improve the accuracy of circRNA functional similarity calculations.

## Data Availability

The Python source codes and the datasets in this work are freely available in the GitHub (https://github.com/CJNabla/MultiSourcCFS).

## Abbreviations

circRNAs: Circular RNAs
GO: Gene Ontology
RBPs: RNA binding proteins
DAG: Directed acyclic graphs
PSSM: Position specific scoring matrix
CMS: CircRNA–miRNA similarity matrix
PCC: Pearson’s correlation coefficient
